# A Fast-Start Pacing Strategy Speeds Pulmonary Oxygen Uptake Kinetics and Improves Supramaximal Running Performance

**DOI:** 10.1371/journal.pone.0111621

**Published:** 2014-10-31

**Authors:** Tiago Turnes, Amadeo Félix Salvador, Felipe Domingos Lisbôa, Rafael Alves de Aguiar, Rogério Santos de Oliveira Cruz, Fabrizio Caputo

**Affiliations:** Human Performance Research Group, Center for Health and Sport Science, Santa Catarina State University, Florianópolis, Brazil; University of Rome, Italy

## Abstract

The focus of the present study was to investigate the effects of a fast-start pacing strategy on running performance and pulmonary oxygen uptake (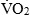) kinetics at the upper boundary of the severe-intensity domain. Eleven active male participants (28±10 years, 70±5 kg, 176±6 cm, 57±4 mL/kg/min) visited the laboratory for a series of tests that were performed until exhaustion: 1) an incremental test; 2) three laboratory test sessions performed at 95, 100 and 110% of the maximal aerobic speed; 3) two to four constant speed tests for the determination of the highest constant speed (HS) that still allowed achieving maximal oxygen uptake; and 4) an exercise based on the HS using a higher initial speed followed by a subsequent decrease. To predict equalized performance values for the constant pace, the relationship between time and distance/speed through log-log modelling was used. When a fast-start was utilized, subjects were able to cover a greater distance in a performance of similar duration in comparison with a constant-pace performance (constant pace: 670 m±22%; fast-start: 683 m±22%; *P* = 0.029); subjects also demonstrated a higher exercise tolerance at a similar average speed when compared with constant-pace performance (constant pace: 114 s±30%; fast-start: 125 s±26%; *P* = 0.037). Moreover, the mean 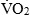 response time was reduced after a fast start (constant pace: 22.2 s±28%; fast-start: 19.3 s±29%; *P* = 0.025). In conclusion, middle-distance running performances with a duration of 2–3 min are improved and 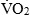 response time is faster when a fast-start is adopted.

## Introduction

The pattern of speed (*s*) distribution chosen during an exercise bout, i.e. pacing strategy, has been shown to have important implications for both activation and proportional contribution of oxidative metabolism to energy turnover [1]. The rationale behind this phenomenon is that the rate of increase in oxygen uptake at the exercise onset (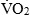 kinetics) is proportional to the rate of phosphocreatine breakdown in active muscles per unit change in time (i.e. the Δ[PCr]/Δt ratio) [2]. In this sense, adopting a higher initial speed during a fast-start pacing strategy (FS) is thought to increase Δ[PCr]/Δt ratios. This enhanced aerobic contribution during the first few seconds of exercise spares an equivalent amount of the anaerobic capacity that can then be used to improve exercise performance [3]. Accordingly, the pacing strategies employed to achieve times within two percent of the world record time in the 800-m track event in international athletics competitions demonstrate that a relatively fast-start over the initial 200 m is the preferred strategy for running performance [4].

Although non-running studies have indicated that a FS improves high-intensity exercise performance by increasing the speed of relatively slower 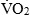 kinetics [mean response time (MRT) approximately 40–50 s] [1], [3], [5], high-intensity running exercises already possess a fast 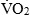 response [6]–[8], which may not increase to an extent that affects performance. Presently, the only indirect evidence on this topic comes from Sandals *et al*. [4], who demonstrated that middle-distance runners attained a lower peak 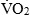 during a constant speed 800-m pace time-to-exhaustion on a treadmill in comparison with a race simulation involving acceleration to a faster speed followed by a speed decline (i.e. FS). Despite the higher 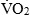 peak indicating a likely higher aerobic contribution, 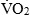 kinetics and total O_2_ consumed was not measured by Sandals *et al*. [4]. Consequently, the actual effect of FS on the overall 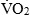 response during supramaximal running performance is still unknown.

A testing protocol was designed to investigate the effects of a FS on aerobic metabolism and performance based on the highest constant speed (HS) that still allows achieving maximal oxygen uptake (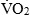
max) during treadmill running. This is an important aspect of this study, since Sandals *et al*. [4] used running speeds that were not able to elicit 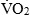
max. Furthermore, HS is also a physiological index representing the constant running speed at which 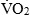
max is reached with the fastest 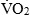 kinetics [9], adding potential concerns about the effects of a FS on metabolic control. Theoretically, the HS would be a suitable intensity in the evaluation of the effects of a FS on middle-distance performance and physiological responses because the physiological determinants of the HS are probably similar to those responsible for middle-distance running performance (i.e. an integrative contribution of aerobic and anaerobic energy systems) [10]–[12]. To predict equalized performance values for a constant pace that can be readily compared to those derived from a FS performance, the relationship between time (*t*) and distance (*d*) or *t* and *s* through log-log modelling from a series of time-to-exhaustion tests was used. For times to exhaustion in the 1–10-min range, the log-log model has been shown to be appropriate and superior to the critical-power model [13].

The focus of the present study was: 1) to compare performance parameters (i.e. distance covered and time-to-exhaustion) using a FS with those predicted from the log-log modelling for constant pace performance; and 2) to assess the effect of a FS on the aerobic contribution during running exercise crossing the upper boundary of the severe-intensity domain [9], [14]. It was hypothesized that a FS would increase performance during supramaximal treadmill running exercises by allowing VO_2_max to be achieved more rapidly during the bout.

## Methods

### Subjects

Eleven active male subjects (28±10 years, 70±5 kg, 176±6 cm, 57±4 mL/kg/min) volunteered for this study. All participants were apparently healthy, non-smokers, free from injury, not taking any medication, and participating in physical activity at least three times a week. Before commencing the study, all participants were informed of the proceedings but remained naive to the study rationale. Subjects were also instructed to avoid strenuous exercise in the 24-h period preceding a test session and to arrive at the laboratory in a rested and fully hydrated state. All volunteers gave written informed consent to participate in this study, which had been approved by the Santa Catarina State University Research Ethics Committee. This work was performed in accordance with the principals of the Declaration of Helsinki.

### Experimental design

**Figure 1:**
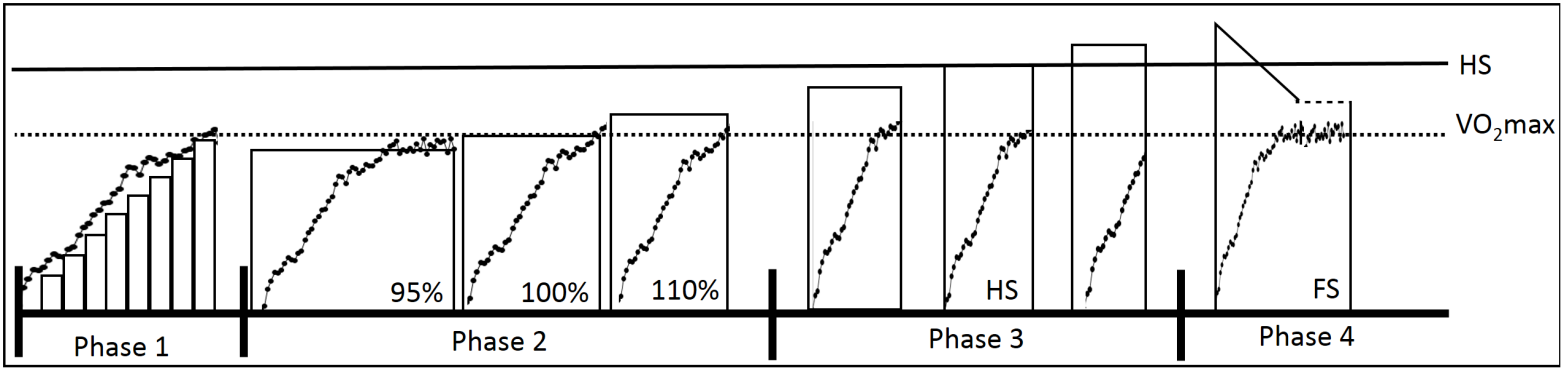
Schematic representation of the protocol timing during the four phases. The superimposed data points are merely illustrative data representing 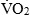 response during tests. 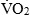
max, maximal oxygen uptake (dashed line); HS, highest speed (solid line); FS, fast-start pacing strategy. See “Methods” for more details on phases one, two, three and four.

Subjects visited the laboratory for four phases of experimentation within a 3-week period, with at least 48-h separating each visit (Figure 1). All tests were performed at the same time of day (±2 h) on a motorized treadmill (Inbramed Millenium Super ATL, Porto Alegre, Brazil) set at a 1% gradient. The four phases of the study comprised: 1) an incremental test in order to determine 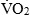
max, maximal aerobic speed and the speed associated with lactate threshold; 2) three laboratory test sessions for the determination of the relationship between *t*×*d*, *t*×*s*, and additional values of 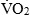
max; 3) the determination of the HS from two to four constant speed tests; and 4) an exercise to exhaustion phase using a higher initial speed followed by a subsequent decrease in speed (i.e., FS protocol). During all tests, subjects were blinded to the time elapsed during exercise and encouraged to continue for as long as possible until volitional exhaustion.

For phases two, three and four, the tests were preceded by a warm up consisting of 10 min of continuous running at the speed of lactate threshold followed by a 5-min rest period. This warm-up was employed because prior moderate-intensity exercise has been demonstrated to have no influence on 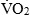 kinetics during subsequent severe-intensity running [15]. All the transitions from rest to running were performed by the participants using the support rails to suspend their body above the belt while they developed cadence in their legs. Time-to-exhaustion measurements started when the participant released the support rails and started running on the treadmill belt.

Throughout each test, respiratory gas exchange was measured breath-by-breath using an automated open-circuit gas analysis system (Quark PFTergo, Cosmed Srl, Rome, Italy). Prior to each test, gas analysers were calibrated using ambient air and gases containing 16% oxygen and 5% carbon dioxide. The turbine flow meter used for the determination of minute ventilation was calibrated with a 3-L calibration syringe (Cosmed Srl, Rome, Italy). For phases one and two, 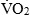 was reduced to 15-s average values and the highest 15-s 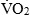 value of each test was used to calculate subject’s 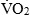
max. For the remaining phases, the achievement (or not) of 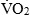
max was calculated based on the highest 15-s rolling average [9].

#### Phase one - Incremental test

The initial treadmill speed was set at 8 km/h and was increased by 1 km/h every 3-min until subject exhaustion. At the end of each stage, a 30-s rest period was required in order to collect capillary blood samples (25 µL) from the non-hyperaemic earlobe in order to measure blood lactate concentration. The speed associated with the lactate threshold was defined as the speed maintained during the stage prior to which the first sudden and sustained increase in blood lactate above the baseline level was observed. The maximal aerobic speed was calculated according to the method of Kuipers *et al*. [16] as the final speed achieved during the test. All subjects fulfilled at least two of the following three criteria for achieving 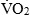
max during the incremental test: 1) respiratory exchange ratio greater than 1.1; 2) a blood lactate concentration greater than 8 mmol/L; and 3) a peak heart rate at least equal to 90% of the age-predicted maximal.

#### Phase two - Predictive trials and 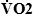
max

On separate days and in a random order, each participant performed three constant speed tests at 95, 100 and 110% of the maximal aerobic speed. The time-to-exhaustion was measured to the nearest second of the subject’s exhaustion. 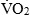
max was then calculated for each subject by averaging the four 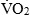
max values obtained during the incremental test and the three predictive trials. The total error in the measurement of 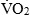
max was also calculated for each subject from the same data as a coefficient of variation (%) [9].

#### Phase three - HS determination

Subjects performed between two and four constant speed tests to exhaustion in order to determine the HS. To ensure whether subjects had (or had not) attained 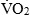
max during these tests, the following criterion was adopted: the maximal 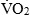 value (calculated as the highest 15-s rolling average) reached in each test should be within the total error of measurement obtained for each subject during 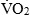
max determination [9]. In the first test, speed was calculated to result in exercise exhaustion within 120 s (as described below). If 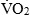
max was attained, further subsequent tests at a 5% higher speed were performed on separate days until 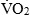
max could not be reached. Conversely, if during the first constant speed test 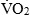
max was not reached, further tests were conducted with reduced speeds (5%) until 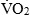
max had been elicited.

#### Phase four – Fast-start strategy protocol

Finally, subjects performed a FS protocol, in which the initial speed was set 10% above the HS and then decreased progressively throughout the test until reaching 90% of the HS at an exercise duration and distance matched to those performed at the HS (Figure 1). The speed of the treadmill was then maintained at 90% of the HS until voluntary exhaustion of the subject.

### Data analysis procedures

#### Log-log modeling

Predicting the intensity that would be expected to lead to exhaustion in 120 s was performed by fitting the predictive trials (*t* and *s*) with a least-squares straight line to the natural logarithms (log-log predictions) for each subject. The performance parameters for constant-pace running were also derived by log-log predictions (*t* vs. *d* or *t* vs. *s*), but using both the predictive trials and the HS. Log-log modelling has demonstrated good reliability in predicting time-trial performance over race-specific distances and seems to be a better predictor in comparison with the critical-power model [13], [17]. Each runner’s times for the standard competition distance of 800-m were also predicted using both strategies. 800-m performance using a FS was predicted by calculating the amount of the intercept used in the extra-time, assuming that the FS does not change the slope of the relationship between *t* and *d*. Measures of goodness of fit for each set of four runs were the adjusted correlation coefficient (square root of the R^2^ adjusted for degrees of freedom) and the standard error of the estimate (SEE).

#### 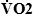 responses

To avoid being influenced by the amount of data used in the comparison between the FS and HS, all of the following calculations, except maximal accumulated O_2_ deficit (MAOD), were analysed to individually fix the time window to the shortest time to exhaustion recorded for each subject (i.e. iso-time).

Occasional errant breath values were removed from the data set if they fell more than three standard deviations outside the local mean (i.e. five-point rolling mean), and the integral area under the 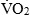 curve representing the total amount of O_2_ consumed during exercise was calculated (OriginPro 8, OriginLab, Massachusetts, USA). Thereafter, to characterize the 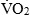 kinetics during the HS and FS, we calculated the MRT for 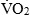 by fitting a mono-exponential curve to the raw data from the onset of exercise using iterative nonlinear regression procedures:
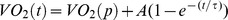
where 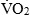
(t) is 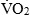 at time t, 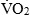
(p) is the pre-test 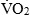; A is the asymptote of the increase in 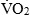 above the pre-test value and τ is the time constant (equivalent to the MRT in this model). For the measurement of 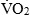
(p), the participant remained standing on the treadmill belt for 5 min prior to the test and the 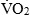 of the last two minutes were averaged. With only one transition performed in each condition, more complex models were not considered suitable [3]. In addition, because the two protocols resulted in the rapid attainment of the 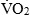
max, a single exponential function starting at the onset of exercise was considered the most appropriate approach for characterizing the overall MRT [18].

The energy cost of running (i.e. the accumulated O_2_ demand) was set in this study as 0.192 mL O_2_ per kg of body mass per meter by using the average value reported by di Prampero *et al.* [19] and correcting for the 1% treadmill gradient [20]. The intercept representing the energy cost at rest (5.1 mL/kg/min) comes from Medbo *et al.* [21]. The MAOD for each condition was estimated by subtracting the total amount of O_2_ consumed from the calculated O_2_ required.

### Statistical Analysis

Calculations were performed with the aid of a spreadsheet for straightforward crossover trial analysis [22]. When no comparisons were involved, the means and between-subject standard deviations were derived from the raw values of the measures; for all other measures, they were derived by performing back-transformation of the log-transformed values and the standard deviations were presented as percentages. Data reliability was assessed by means of the retest correlation (intraclass correlation coefficient; ICC) and the measurement errors (typical error or SEE) along with 90% confidence limits. The inflated typical errors were reported because there were no identifiable individual responses to the treatment. Uncertainties in the measurement errors are presented as factors. To make inferences about true (population) values of the effect (%) of a FS on performance and physiological responses, the uncertainty in the effect was expressed as 90% confidence limits and as likelihoods that the true value of the effect denotes real positive (+ive) or negative (−ive) change; this was represented by the probability (*P*) value derived from the t statistic followed by qualitative interpretation [23]. To evaluate the relationship between performance variables and to assess the association between performance and a set of physiological variables, single and multiple (stepwise) linear regressions analyses were used, respectively.

## Results

In the incremental test, subjects attained a maximal aerobic speed of 16.1±1.8 km/h and the speed at lactate threshold was 9.8±2.7 km/h. The time-to-exhaustion for exercise at 95, 100 and 110% of the maximal aerobic speed was 561±143 s, 369±82 s and 214±72 s, respectively. The calculated subject’s 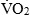
max was 3982±429 mL/min. The individual error in the measurement of 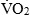
max (i.e. the coefficient of variation of the four 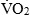
max values) ranged between 0.7 and 7.8% (mean ± SD of 3.0±2.3%). During the third phase of the experiment, subjects attained a HS at 20.1±2.0 km/h (time-to-exhaustion of 108±34 s), representing 126±13% of maximal aerobic speed.

The adjusted correlation coefficients for the log-log modelling of the sets of four runs were all at least 0.999 for the relationships between *t*×*d* and averaged 0.993 (SD of 0.008) for the relationship between *t*×*s*. The application of the models revealed a very large correlation between the HS and predicted constant-pace 800-m performance [r = −0.80 (–0.93 to −0.43)]. Stepwise multiple regression analyses further demonstrated that the major predictors of both the HS and predicted 800-m performance were, in order of importance, relative MAOD, relative 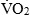
max and MRT. The increase in multiple correlation coefficients with the addition of each predictor was 0.59–0.81–0.89 for the HS and 0.59–0.88–0.89 for the predicted 800-m performance. The upper and lower 90% confidence limits for the full models were identical: 0.68–0.96.

**Figure 2:**
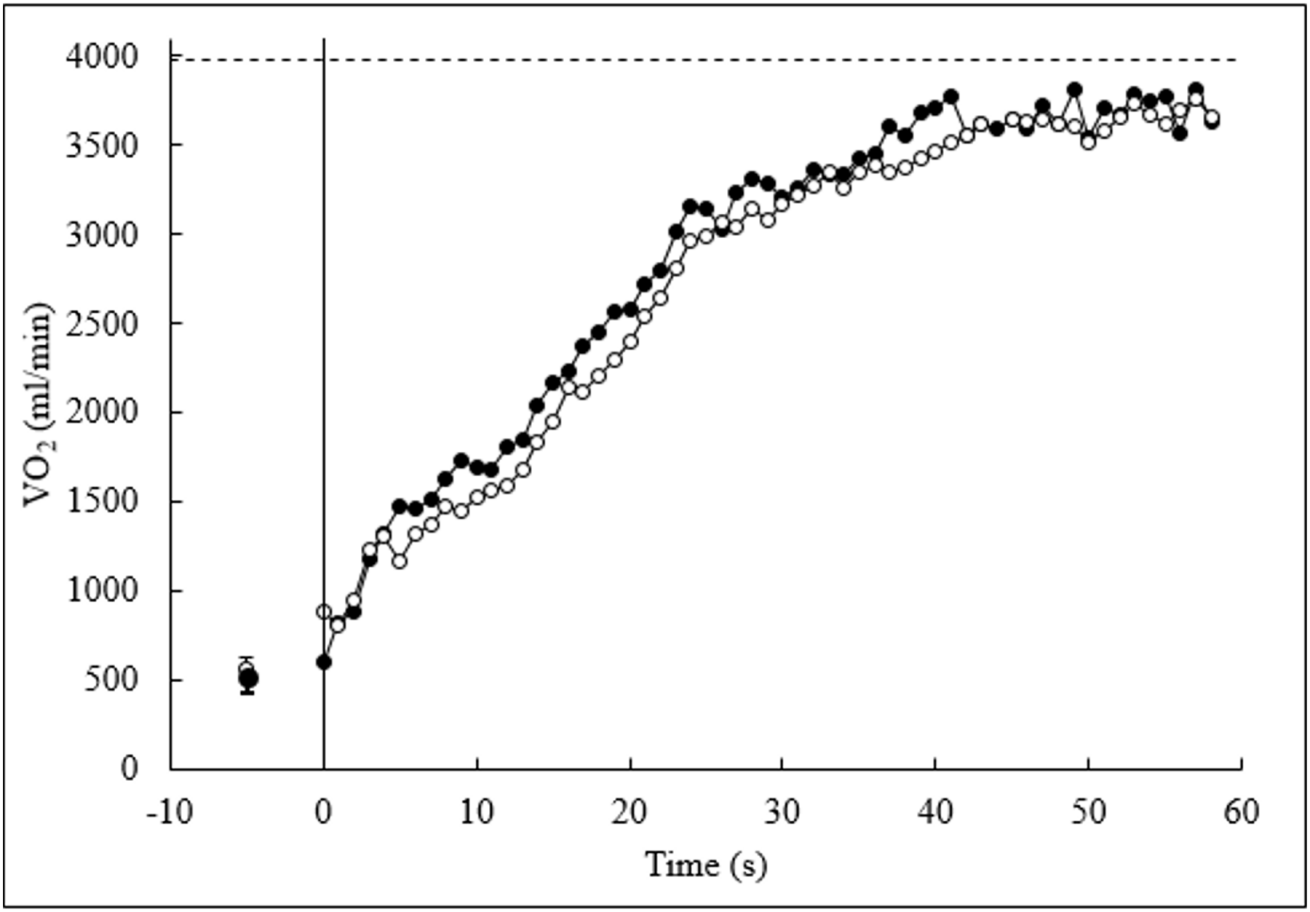
Group mean pulmonary 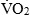 response during the highest speed and fast-start pacing strategy. For graphical presentation, data were matched at the shortest time to exhaustion recorded and interpolated to show second-by-second values. The vertical solid line represents the onset of exercise and the horizontal dashed line is the mean 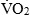
max. The mean ± SD of pre-test 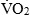 in each condition are also shown.

Table 1 shows the comparison of the various performance parameters obtained during the FS with the approximations derived by the models for constant pace exercise. The benefit of the FS was very likely for all performance variables. In addition, the performance improvement was also very likely beneficial when comparing the predicted 800-m performance using both strategies. The 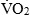 responses observed during the HS and FS performances were compared at iso-time and iso-distance (Figure 2 and Table 2). The FS very likely reduced the MRT and increased the amount of O_2_ consumed. There was no clear difference in MAOD between the experimental conditions. Moreover, the accumulated O_2_ deficit spared at iso-time with the FS (145±179 mL) was quite similar to that used to maintain the exercise during the FS after the iso-time (134±185 mL).

**Table 1:** Comparison analysis of the FS performance variables with those predicted for constant pace from the log-log modelling.

	Mean ± coefficient of variation (%)						
Performance Measures	Constant Pace	FS	Correlation and 90%CL	SEE (%)^a^	% changes ±90%CL	*P* value	Qualitative Inference
Exercise tolerance at mean FS speed (s)	114±30	125±26	0.89 (0.71–0.96)	8	9±7	0.037	Benefit very likely
Time to cover FS distance (s)	128±26		0.99 (0.98–1.00)	1.5	−2.5±1.8	0.033	
Distance covered at FS duration (m)	670±22	683±22	0.99 (0.98–1.00)	1.3	2.0±1.4	0.029	
Predicted 800-m^b^ (s)	155±11	152±10	0.97 (0.90–0.99)	1.5	−2.0±1.6	0.046	

Data are back-transformed means ± coefficients of variation.

a Uncertainties in these errors: ×/÷ 1.2. Multiply and divide the error by this number to obtain the 90% confidence for the true error.

b The 800-m using a FS was predicted by calculating the amount of the intercept used in the extra-time assuming that the FS does not change the slope of the relationship between *t* and *d*.

FS: fast-start pacing strategy.

**Table 2:** Observed changes in physiological responses after a FS in comparison with constant speed exercise.

	Mean ± coefficient of variation (%)						
Physiological Measures	HS	FS	Correlation and 90%CL	Inflated Error^a^ (%)	% changes ±90%CL	*P* value	Qualitative Inference^b^
Pretest 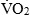 (mL/min)	555±14	511±18	0.51 (0.02–0.80)	11	−8±8	0.095	Very likely –ive
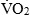 Mean Response Time (s)	22.2±28	19.3±29	0.80 (0.50–0.93)	13	−13±8	0.025	Very likely –ive
Amplitude (mL/min)	3396±12	3419±8	0.74 (0.39–0.90)	5.5	0.7±4.1	0.769	Unclear
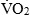 max (mL/min)	3871±10	3874±8	0.93 (0.80–0.98)	2.6	0.1±2.0	0.941	Unclear
O_2_ consumed at iso-time (mL)	5373±45	5503±46	1.00 (0.99–1.00)	2.6	2.4±2.0	0.051	Very likely +ive
MAOD (mL)	2385±34	2425±30	0.89 (0.70–0.96)	11	2±8	0.713	Unclear

Data are back-transformed means ± coefficients of variation.

a Uncertainties in these errors: ×/÷1.5. Multiply and divide the error by this number to obtain the 90% confidence for the true error.

b The effect was deemed unclear if the chances that the true effect has the same sign than that of the observed effect were lower than 75%.

FS: fast-start pacing strategy; HS: highest constant speed; MAOD: maximal accumulated O_2_ deficit.

## Discussion

Non-running studies of similar duration, most of them conducted in cycling, reported that improvements in time trials or time-to-exhaustion performances with a FS are usually accompanied by faster 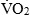 kinetics and higher O_2_ consumption for a given time [1], [3], [5]. The results of this study are in concordance with these previous reports demonstrating the benefits of a FS in comparison with more conservative pacing strategies during treadmill running. In spite of the lower magnitude of MRT reductions, which were not correlated with change in performance in the present study, the faster achievement of 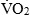
max induced by the FS increased the aerobic contribution even in an already fast 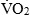 response. This seems to have resulted in a spared quantity of the anaerobic capacity, measured in the present study by the oxygen deficit. This quantity was equivalent to those used to prolong the exercise tolerance at a running intensity correlated with that of 800-m performance. Consequently, these results are in accordance with the established models of mitochondrial respiratory control, in which changes in muscle [PCr], [ADP] and [Pi] per unit change in time are responsible for mitochondrial respiratory control through the rate of oxidative phosphorylation in the active muscles during exercise [24], [25].

The approximations derived from the log-log modelling for changes in time trial performance in the present study (2.5%) are within the effects usually reported for human performance experiments where the end-point is known. Although our subjects were not competitive runners, the significance of this effect in terms of magnitude should be discussed from a practical perspective. Hopkins *et al*. [26] have demonstrated through simulations, that the increase in the chances of winning an event varies uniformly when a particular subject benefits with an enhancement corresponding to multiples of the within-subject random variation within a group of identical subjects (i.e. between-subject variation of zero, equal to a repeated measures design). Even though a more careful analysis of the reliability of subject’s performance has not been conducted, the typical error of measurement usually lies around 2–3% for groups with similar characteristics [17]. Therefore, we can be confident that the observed enhancement is meaningful for this group of subjects because the ratio between the observed effect and the typical error should increase the chances of winning by approximately 30% in cases where the subject runs against himself in a hypothetical simulated event, which can be considered as a moderate effect [30]. For the log-log predictions of 800-m performance the benefits of a FS decreased slightly, yet were still meaningful, probably as a consequence of the amount of anaerobic energy spared with the higher aerobic contribution becoming relatively lower in comparison with the total energy cost as the time/distance increases.

Although the present study has demonstrated that a FS enhanced performance by speeding an already fast aerobic response to exercise in non-athletes, extrapolating these results to middle-distance runners is an interesting issue. Athletes present even faster 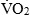 kinetics for a given running speed and obviously have higher absolute speeds during performances of similar distance [27], [28], which could prevent meaningful accelerations in 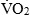 kinetics. Indeed, Thomas *et al*. [29] observed that elite runners reached 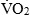
max in a very fast time during an 800-m race (around 45 s). Conversely, it was demonstrated that the time to reach 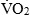
max in runners is liable to be reduced as a function of the initial speed at very high intensities [30]. In addition, athletes have higher values of 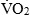
max relative to body mass and, consequently, they may still spare an important amount of energy, although with a lesser acceleration in 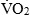 kinetics. In other words, since the athletes present higher values of 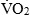 along the transition from rest to exercise, a lower effect of the FS in the speed of 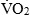 kinetics may not be a problem because the absolute amount of energy spared would be similar between athletes and non-athletes [27]. Therefore, although it is recognized that athletes generally present lower performance improvements in terms of magnitude than non-athletes for a given intervention, it is hypothesized that they would also benefit from a FS, since enhancements as low as 0.5% are considered important to elite runners [31], [32].

The linear regression analysis, irrespective of being single or multiple, demonstrated that both the HS and 800-m speeds are linked with each other and are highly influenced by the same physiological parameters. There is no novelty in the fact that success with middle-distance running is dependent on an integrative contribution from the aerobic and anaerobic variables that allow a runner to maintain a rapid velocity during a race [10]–[12], and the results of this study are consistent with the notion that having fast 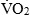 kinetics is also important. Similarly, it is intuitive that a large anaerobic capacity allows for greater endurance at any given intensity and thus to continue reaching 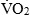
max at higher relative intensities [33]. Therefore, large anaerobic energy stores combined with a high aerobic power should yield a higher HS, especially when allied to a fast 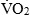 kinetics. Therefore, it is hypothesized that the HS may group together several intervening factors for middle-distance performance into a single physiological index, which has proven sensitive to high-intensity training in an ongoing study (unpublished observations).

One possible limitation of the present investigation is the lack of randomization between the HS and FS. While the nature of the present experiment rendered it impossible to control any possible order effects, the subjects were mostly accustomed with exercising to exhaustion. Furthermore, it is likely that three predictive trials plus two-to-four tests for the determination of the HS, the latter of which were at or very close to the HS and FS intensities, provided enough familiarization to prevent learning effects in the last two non-randomized trials. Conversely, if the high number of tests performed until exhaustion had caused an accumulated fatigue in the subjects, the observed result should be the opposite to what was found in the present study if there was no systematic effect of the FS on performance. Therefore, it is unlikely that any order effects influenced these findings.

In conclusion, the results generated suggest that running performance over 2–3 min is improved when a FS is adopted. The higher aerobic contribution resulting from faster 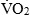 kinetics in the early phase of exercise spares an important amount of the finite anaerobic capacity, which can be used as an additional energy source to improve middle-distance performance. It is recommend that future studies investigate how these effects would behave/interact in the presence of other strategies that are commonly used to speed 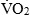 kinetics such as prior exercise.
